# An Evaluation of Whole-School Trauma-Informed Training Intervention Among Post-Primary School Personnel: A Mixed Methods Study

**DOI:** 10.1007/s40653-021-00432-3

**Published:** 2022-01-05

**Authors:** Justin MacLochlainn, Karen Kirby, Paula McFadden, John Mallett

**Affiliations:** 1 School of Psychology, Ulster University, Cromore Road Coleraine campus, Co. Derry, Coleraine, BT52 1SA Northern Ireland; 2 School of Applied Social and Policy Sc. Institute for Research in Social Sciences, Magee campus, Derry, BT48 7JL Northern Ireland

**Keywords:** Whole-school, Trauma-informed, Education, Burnout, Secondary traumatic stress, Teacher, Wait-list control, Compassionate schools

## Abstract

Students’ ability to reach their potential in school—both behaviourally and academically – is linked to their educator’s knowledge of child and adolescent development, childhood adversity and trauma, and how these impact learning and behaviour. However, teacher pre-service training programmes often offer inadequate instruction to meet the needs of trauma-impacted students. The purpose of the study was to investigate the benefits of professional development training in trauma-informed approaches on school personnel attitudes and compassion fatigue. There is a paucity of research on whole-school trauma-informed approaches and most have methodological limitations via the absence of a control group. In addressing this gap, the study is one of the first to utilise a control group in the research design to ensure findings are robust. The study utilised a quasi-experimental wait-list control pre-post intervention design to evaluate the efficacy of trauma-informed professional development training. We compared attitudes and compassion fatigue among 216 school personnel (*n* = 98 intervention, *n* = 118 comparison) utilising the Attitudes Related to Trauma-Informed Care (ARTIC) scale and the Professional Quality of Life scale (Pro-QoL). Quantitative data was supplemented by qualitative focus group data. Findings demonstrated that school-personnel within the intervention group reported significant improvements in attitudes related to trauma-informed care, and a significant decrease in burnout at 6-month follow-up. Our findings demonstrate that with minimum training on the dynamics of trauma, personnel attached to a school can become more trauma-informed and have more favourable attitudes towards trauma-impacted students and consequently be less likely to experience burnout.

## Introduction

Neurodevelopmental science has demonstrated how exposure to childhood adversity such as abuse (i.e., sexual, physical, and psychological), neglect (i.e., physical, and emotional), household dysfunction (i.e., parental mental illness, substance misuse, domestic violence, and criminality) and adverse social environment categories such as bullying, discrimination, and socio-economic deprivation can significantly alter the child’s ability to engage with classroom activities (Bradshaw et al., 2012). These Adverse Childhood Experiences (ACEs) and other stressful events (i.e., illness, loss, and having close friends experiencing psychological difficulties) are closely linked to a multitude of psychological and physical problems in adulthood and additionally to concurrent problems with emotional, social, and cognitive development leading to a range of behavioural and psychological difficulties (Clark et al., 2010; Enlow et al., 2012; Felitti & Anda, 2010; MacLochlainn et al., 2021; McLaughlin et al., 2014).

Toxic stress from repeated and prolonged ACEs and other stressful events is theorised to be the primary driver of these problems and consequently has an enduring effect on the child’s brain development (Burke et al., 2011; Shonkoff et al., 2012). Children who experience toxic stress show signs of problems relating to the developmental areas of executive functioning such as attention, complex planning, impulse control, decision making, working memory, and social and behavioural modulation, extending to problems in emotional regulation, impulsivity, and communication (Cook et al., 2017; Shonkoff et al., 2012; Steiner, 2016; Van Dam et al., 2018). Indeed, and as the extant literature suggests, ACEs and other stressful events may contribute to low academic attainment, and conduct problems, leading to an increased risk of suspension, expulsion, absenteeism, and risky behaviours (Bellis et al., 2018; Delaney-Black et al., 2002; Ford et al., 2006).

Owing to this increased risk for impulsivity and conduct problems during the school day, some trauma-impacted students are often in conflict with their teachers (Fazel et al., 2014). Difficult teacher/student relationships may in part be due to miscommunication stemming from the educator’s limited understanding of a child’s lived experience of trauma and its impact on teaching and learning (Gray et al., 2017; Wilson, 2019). Additionally, many staff with their own high level of ACEs and trauma history may be re-traumatised by student behaviour with teachers reporting that stress resulting from students’ disruptive behaviour being central to experiences of burnout and reasons for leaving the profession (Fazel et al., 2014).

In schools across the UK and Ireland, teachers possess a limited understanding and awareness of the impact of trauma on student learning and behaviour and how to mitigate misbehaviour in the classroom (McKee & Dillenburger, 2009; Sitler, 2009). Teacher pre-service training programmes often offer inadequate instruction to meet the needs of trauma-impacted students (Brunzell et al., 2018; McInerney & McKlindon, 2014). Omitting adequate instruction from training programmes may result in teachers having a deficit in knowledge and skills and consequently developing challenging relationships with their students. Due to this lack of knowledge, students are assumed as being problematic, delinquent, or truant rather than vulnerable and in need of additional supports (Cole et al., 2013; Dorado et al., 2016; Moore et al., 1997).

Fortunately, and with the groundswell of ACE literature linking childhood trauma to a range of both proximal and distal negative outcomes, child welfare advocates have begun to encourage the implementation of whole-school trauma informed practice (Thomas et al., 2019; Wolpow et al., 2009). Practice is viewed as being trauma informed providing it promotes healthy, caring, and supportive relationships between students, teachers, and ancillary staff (Parker et al., 2019). Parker et al. (2019) outline the benefits of nurturing relationships highlighting an increase in resilience, self-regulation, executive function, and interpersonal competence in traumatised youth. Trauma informed practice requires a paradigm shift in perspective and attitude illustrated by not asking “*What is wrong with you*?” when a problematic behaviour occurs but rather asking /exploring “*What has happened to you?*” instead (Wolpow et al., 2009). This paradigm shift involves a refocus on understanding what has happened or is happening in the child’s life, rather than merely focusing on the behaviour (Kenny et al., 2017; Weare, 2015). Trauma informed approaches embody a holistic framework to realign organisational culture, policies, and practices to be aware of and sensitive to the desire to help alleviate pain and foster healing of traumatised individuals (McInerney & McKlindon, 2014; Webb et al., 2020).

Clinical research suggests that increasing staff’s knowledge and understanding of trauma and trauma informed practice leads to more positive attitudes towards trauma informed approaches (Brown et al., 2012). However, the bulk of trauma-informed evaluation research has been produced within education organisations in the US where provisions for trauma-informed practice were legislated for via the: *Every Student Succeeds Act* (ESSA, 2015). Whilst the US has embraced whole school trauma informed approaches in schools, there is still a dearth of robust methods of evaluation; for example, no studies utilised a control group in their evaluations. Within the UK there has also been considerable interest in cultivating trauma-informed approaches across various systems and service settings (Bunting et al., 2019). In relation to schools, Education Scotland (2018) has espoused the need to integrate trauma-informed principles into already established frameworks such as the Nurture approach which is a relational-based programme to support children and young people through a small number of trained personnel within the school (Education Scotland, 2018).

Another UK school-based programme that implemented trauma-informed approaches in education is the Attachment Aware Schools Programme (Fancourt & Sebba, 2018). This professional development programme better equips trained staff to meet the emotional needs of their students, however, as with the Nurture approach, training was only provided to a small number of staff within each participating school (Dingwall & Sebba, 2018; Fancourt & Sebba, 2018). However, evaluations of these studies have identified areas to be targeted for the effective implementation of trauma-informed practice in schools. One specific area of recommendation was the professional development of all school personnel (not just teachers) as all school staff are involved in responding to behaviour (Dingwall & Sebba, 2018). This recommendation was in line with a recent meta-analysis indicating that interventions yield most successful outcomes when adopting a whole-school approach (Goldberg et al., 2019).

To our knowledge, within the UK and Ireland, only one study has been published on whole-school trauma-informed approaches within educational settings. Barton and colleagues (2018) piloted an ACE-informed whole-school approach as a feasibility study in 3 primary schools in Wales (Barton et al., 2018). Despite the absence of a control group, the study highlighted the positive impact trauma-informed training can have on teaching staff. This gap in the literature offers this study the unique opportunity to evaluate the training of a whole-school trauma-informed approach in a post-primary school in N.I. In addressing this gap, the study will be one of the first to utilise a control group in the research design to ensure findings are robust. Notwithstanding the dearth of robust research designs evaluating the implementation of trauma-informed care (TIC) approaches within the education system, the literature does demonstrate that TIC approaches can increase staff awareness of the impact of trauma, change staff perspectives and attitudes towards trauma-impacted students, and potentially lead to improved staff well-being (Kim et al., 2021; Plumb et al., 2016).

### Rationale

There is a growing concern related to high teacher attrition with research indicating that 25% of new teachers leave the profession in their first year (Aloe et al., 2014). Specifically, stress resulting from being re-traumatised, being ill-equipped to deal with students’ disruptive classroom behaviour, and lack of support systems within schools, have been highlighted as major factors leading to teacher burnout and reasons for leaving the profession (Fazel et al., 2014). Therefore, there is an urgent need to intervene to reduce re-traumatising and burnout in school staff, to increase staff understanding and awareness of the impact of trauma on student learning and behaviour and how to mitigate misbehaviour in the classroom, and to implement self-care and community-care strategies to assist school staff in their daily duties.

## Aims and Objectives

The overall objective of the study was to introduce all school personnel to trauma-informed practices to support vulnerable young people and to aid in the wellbeing of staff.

Therefore, the aims of the current study were to determine:

1. whether a 2-day professional development training (workshop) in trauma-informed approaches would change school personnel attitudes related to trauma-informed care post-workshop and if any changes made were maintained at 6-month follow up.
2. whether the workshop influenced school personnel levels of compassion fatigue, e.g., burnout, and secondary traumatic stress (STS), and levels of compassion satisfaction (CSAT) at 6-month follow up.

## Hypotheses

1. It was expected that school personnel attitudes related to trauma informed care would increase post-workshop and this increase would be maintained at 6-month follow up.
2. It was expected that levels of compassion satisfaction (CSAT), secondary traumatic stress (STS), and burnout of school personnel would significantly improve following implementing trauma informed practice within the school.

## Design and Participant Sample

**Fig. 1:**
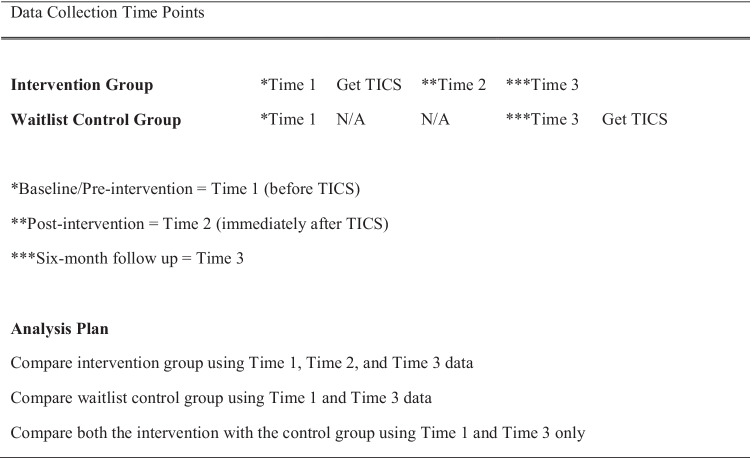
Design and Data Collection Time points

The study utilised a quasi-experimental wait-list control pre-post intervention design to evaluate the efficacy of a 2-day trauma-informed care workshop in one post-primary school in the Northwest region of Northern Ireland over a 6-month period (*n* = 98). This school was experiencing high levels of suspensions and expulsions of students and staff were at a loss on how to intervene. Two post-primary schools in N.I (Mid-Ulster region) consented to participate as a waitlist list control group. These schools, with similar demographics to the experimental group, indicated struggling with the rising levels of mental health issues amongst their students and were aware that some pupils had significant social problems. They were approached by the chief investigator (CI) and asked to participate in the study. Both control schools were invited to participate to ensure adequate matching participant numbers. Wait-list control participants (*n* = 118) did not receive the intervention at this time (see Fig. 1). A waitlist control group is preferable to a control group that receives no intervention as ethically it was considered important not to deny participants access to the trauma informed compassionate schools’ workshop (TICS). All school personnel were invited to participate.

## Ethical Approval Procedures

Ethical approval was granted in accordance with regulations in relation to research governance in Ulster University on studies involving human participants (REC.20.0053). Consent to undertake the study within the school environment was initially provided by the school principals. Prior to the commencement of the workshop, all participants in both the intervention group (*n* = 98; age range: 29 – 64 years: *M* = 46.55, *SD* = 7.70) and waitlist control group (*n* = 118; age range: 22 – 66 years: *M* = 41.91, *SD* = 11.02) received an information pack containing a participant information sheet (PIS) outlining the purpose of the study and a consent form to sign. All data provided was anonymous and was treated in a confidential manner and retained in a secure location in line with Ulster University research governance rules on retention and storage of personal data. Participation was voluntary, and individuals were aware they could withdraw from the study at any point without consequences from their employer. In the case of withdraw from the study, the research team agreed not to retain any previous collected data from the withdrawn participant in line with data protection legislation. Within the PIS, participants were advised that if they experienced psychological distress as a result of the study to contact Lifeline, Samaritans, or the Employee Assistance Program which is included in the staff care policy in their school.

## Methodology

### Data Collection

The pre-workshop survey consisted of participants (*n* = 216; see Table 1) completing a series of demographic items, compassion satisfaction (CSAT) and compassion fatigue measures as well as the 35-item version of the Attitudes Related to Trauma Informed Care scale (ARTIC) (see Measures section). Written instruction for completion was printed on each questionnaire, and participants were given verbal instruction by the research team. To ensure confidentiality staff were assigned a unique identifier which was the last four digits of their mobile phone. Demographic information such as age, gender, ethnicity, staff role, duration within the profession, and any previous trauma training were also collected. Immediately following the 2-day workshop, school personnel participating within the intervention group were surveyed again (post-intervention training: time 2, see Fig. 1) to determine any variation in attitudinal change attributed to the workshop (*n* = 75). Finally, both the intervention group and control group were surveyed once more at 6-month follow up (time 3) to determine if any changes were maintained over time (*n* = 65).

**Table 1:** Demographics of Intervention and Control Groups

	*n*	Min	Max	M (SD)
Age	216	22	66	44.01 (9.91)
Intervention	98	29	64	46.55 (7.70)
Control	118	22	66	41.91 (11.02)
		**Intervention** ***n*** **(%)**		**Control** ***n*** **(%)**
Gender				
Male	55	28 (28.6%)		27 (22.9%)
Female	156	70 (71.4%)		86 (72.9%)
Prefer not to say	5	-		5 (4.2%)
Role				
Administration	24	8 (8.2%)		16 (13.6%)
Facilities	8	6 (6.1%)		2 (1.7%)
Student support	15	8 (8.2%)		7 (5.9%)
Teaching Support	169	76 (77.6%)		93 (78.8%)
Duration				
0–2 years	13	3 (3.1%)		10 (8.5%)
3–7 years	28	6 (6.1%)		22 (18.6%)
8–14 years	44	19 (19.4%)		25 (21.2%)
Over 15 years	131	70 (71.4%)		61 (51.7%)
Any previous training				
Yes	32	21 (21.4%)		11 (9.3%)
No	132	63 (64.3%)		69 (58.5%)
Missing	52	14 (14.3%)		38 (32.2%)
Ethnicity				
Caucasian	189	89 (90.8%)		100 (84.6%)
Asian	1	1 (1%)		-
Other	12	3 (3.1%)		9 (7.7%)
Prefer not to say	12	5 (5.1%)		9 (7.7%)

### Teacher Professional Development Workshop

The workshop comprised of psychoeducation surrounding the nature and impact of trauma along with the nature and impact of compassion through the principles and domains of the Compassionate Schools (CS) approach. *The Heart of Learning and Teaching Handbook* (Wolpow et al., 2009) was utilised in this study as the main instructional material. The handbook contains valuable information for teachers who work with trauma-impacted youth daily. This resource was grounded in evidence-based findings from existing resources and programs. CS benefits related to school staff include, increased job satisfaction and performance, increased self-care and well-being, improved ability to apply trauma-informed teaching and increased knowledge of learning architecture and pedagogy (Hertel et al., 2009). The material remains free to use and can be easily integrated into other schoolwide programmes (Anderson-Ketchmark & Alvarez, 2010). The handbook module content presented in the workshop comprised; a) an introduction to trauma-informed compassionate schools programme, b) information on how trauma impacts a child’s ability to learn, c) the goals of a trauma-informed compassionate school, d) self-care guidance for teaching staff, e) trauma-informed classroom strategies, and f) the importance of community engagement. The Chief Investigator assisted by the research team adapted and consolidated the materials into PowerPoint presentations which were delivered by members of the research team trained in TIC.

In addition, the presentation explored the nature and impact of compassion and how behavioural displays of compassion may be a protective factor impacting on student resilience. The scientific and theoretical foundation for Compassionate Schools (CS) is found within the increasing volume of literature on trauma and complex trauma. CS aims to improve social and emotional learning, and academic skills of students, while simultaneously increasing well-being within staff (Hertel et al., 2009). Teacher compassion equates to feelings of empathy and respect for students who experienced trauma and adversity with the intention to alleviate pain and foster healing (Axelsen, 2017). Similar to other models, CS included management consultation comprised of several meetings and culminating in a strategy to modify and adapt the schools’ student behaviour management policy, procedures, and practices towards a more trauma-informed approach. Management consultation remained ongoing for one full academic year.

### Focus Group Interviews

Seventeen members (71% female) of teaching staff including pastoral care staff within the intervention school were interviewed using a semi-structured framework. Focus group interviews were carried out in two sittings lasting more than an hour each for both practical reasons, and to encourage shared reflections on their experiences three months following the professional development training in Compassionate Schools (CS) (Wolpow et al., 2009). A mixed methods approach was selected to give voice to teaching staff and to ensure quantitative findings were grounded in participants’ experiences. Data were analysed using reflective thematic analysis based on Braun and Clarke’s six phase framework (Braun & Clarke, 2006). The data was derived from semi-structured interviews, where the interview schedule was designed to explore teachers’ understanding of childhood trauma, work related changes following training, personal growth, perceived barriers to implementation, self-care, and their suggestions to improve training. Participants were recruited through a direct approach at the school and interviews were carried out on-site. All participants were informed that they would be referred to by pseudonyms in all accounts of analysis to protect their anonymity. Participants were instructed to talk openly and at length on each topic. Focus group interviews were recorded using an iPad video/audio app along with an audio recording device on an android mobile phone app as backup. Interviews were then transcribed verbatim onto MS Word.

### Measures

#### Attitudes Related to Trauma Informed Care Scale (ARTIC-35; Baker et al., 2016)

Evidence of systems being trauma-informed depends on the extent of moment-to-moment, day-to-day behaviour of its personnel (Metz et al., 2007). Attitudes related to trauma-informed care are believed to be a major catalyst of this behaviour (Baker et al., 2016). Based on exploratory research in professional training, behaviour change, and programme implementation, trauma-informed approaches have the potential to transform behaviour by means of knowledge and attitude change. The ARTIC scale is a direct, efficient, and cost-effective measure of attitudes applicable for school staff and other systems assisting trauma-impacted individuals (Baker et al., 2016). Within the intervention group in this study, attitudes related to trauma-informed care were measured at three time-points (see Fig. 1). This psychometric test was designed for use in schools implementing trauma informed interventions. The scale can be used to measure the readiness of school staff to implement trauma informed practice, any barriers present, and attitudinal change following intervention. The ARTIC includes a series of self-reported Likert items scaled from 1 to 7 across five sub-scales including; 1) underlying causes of problem behaviour and symptoms—with a sample item – *Students are doing the best they can with the skills they have*; 2) responses to problem behaviour and symptoms – with a sample item – *Students need to experience real life consequences in order to function in the real world*; 3) on-the job behaviour – with a sample item—*Being upset doesn’t mean that students will hurt others*; 4) self-efficacy at work – with a sample item -*Each day is uniquely stressful in this job*; and 5) reactions to work—with a sample item – *When I feel myself “taking my work home,” it’s best to bring it up with my colleagues and/or supervisor(s),* with higher scores reflecting more positive attitudes towards developing trauma informed practice. In this study the overall scale demonstrated good internal consistency α = 0.87 (time 1) and α = 0.92 (time 3).

#### Professional Quality of Life Scale (ProQol; Stamm, 2010)

People who work in helping professions assist individuals or communities in times of crises. Helpers are located in the health care system, legal system, and educational system (Stamm, 2010). The Professional Quality of Life Scale is the most used scale to measure both the positive and negative effects of assisting trauma-impacted individuals (Stamm, 2010). Within this study, data was collected (see Fig. 1) to measure compassion satisfaction (CSAT) and compassion fatigue (i.e., burnout and secondary traumatic stress) using the Professional Quality of Life Scale (ProQol; Stamm, 2010). The ProQol is a quality that people feel and attribute to their work. Influenced by both positive and negative affect of helping others who have experienced trauma, the ProQol measures compassion satisfaction (CSAT) and compassion fatigue. CSAT relates to the pleasure derived from being able to do your work well. Compassion fatigue was split into two parts – behavioural burnout, represented by exhaustion, frustration, anger and depression, and – secondary traumatic stress (STS), represented by negative feelings driven by fear and work-related trauma (Stamm, 2010). Employing 30 self-report Likert items, scaled from 0 to 5 with 0 = never and 5 = very often, the ProQol is split into three subscales of ten items each addressing dimensions of CSAT – with a sample item, *I feel invigorated after working with those I [help]*; STS – with a sample item, *I find it difficult to separate my personal life from my life as a [helper]*; and behavioural burnout – with a sample item, *I am not as productive at work because I am losing sleep over traumatic experiences of a person I [help].* In this study, the 3 subscales demonstrated good internal consistency with Cronbach’s alpha values in the range (0.74—0.89) at time 1 and (0.74 -0.88) at time 3.

### Data Analysis

#### Quantitative Analysis

Collated data was coded, cleaned, and prepared for analysis and entered onto a password-protected file using IBM SPSS V.25 statistical software. To ensure data accuracy, pre-analysis was conducted to assess any missing data and extreme values within the dataset. A paired sample model was used. To achieve power of 0.95 with alpha at 0.05 with an effect size Cohen’s d = 0.5, G*Power estimated that a sample size of 45 was required to detect a group by time interaction effect. To check the adequacy of fit of the data, tests were conducted to ensure the assumptions of the given statistical procedure were met.

A battery of paired samples *t*-tests were conducted to address research questions 1 and 2. A two-tailed *t*-test was conducted to evaluate the impact of the workshop on staff attitudes about trauma informed care and if any change in attitudes were maintained at 6-month follow up. In addition, paired samples *t*-tests were also conducted to evaluate whether measures of compassion satisfaction (CSAT) and compassion fatigue of all school personnel participating in the workshop changed significantly following implementing trauma informed practice within the school. This analysis was suitable for this study because it established whether there was a significant change in paired means at each time point (Mertler & Reinhart, 2016).

**Fig. 2:**
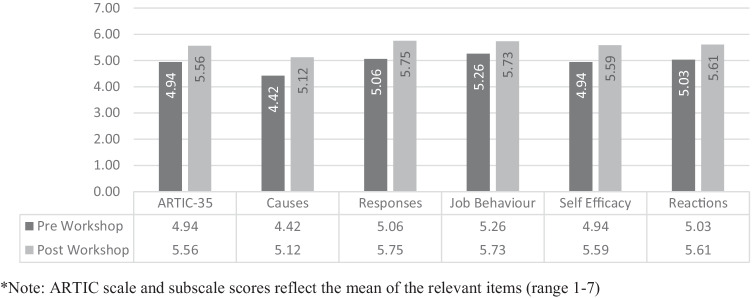
Time1/Time2 means of ARTIC scales pre and post workshop for the intervention group (*n* = 75)

The mean (*M*) and standard deviation (*SD*) of pre-workshop, post-workshop, and 6-month follow up for each of the ARTIC outcome variables are presented in Figs. 2 and 4.

Following this, a series of Mixed repeated measures analysis of variance (ANOVAs) determined whether the mean changes in outcomes from time 1 to time 3 (i.e., 6-month follow up) differed between the intervention and waitlist control group. The independent variable, treatment group, was categorised into two groups i.e., intervention group and waitlist control group. The dependent variables consisted of ARTIC-35, compassion satisfaction, and compassion fatigue.

Mixed ANOVA assumptions were tested satisfying the requirements of *t*-test analysis prior to hypotheses testing (Mertler & Reinhart, 2016).

#### Qualitative Analysis

Focus group data were analysed using reflective thematic analysis based on Braun and Clarke’s six phase framework (Braun & Clarke, 2006) because of its flexible methodology and potential to provide enriched and detailed accounts of data. Following transcription by the first author, the data was printed for further analysis using an inductive approach described by Hayes (2000). The data was repeatedly read searching for meaning and/or patterns before the coding process commenced. The first author began taking notes and marking ideas for coding. The next phase of analysis involved the process of coding and data was organised into meaningful groups. All data extracts were coded and collated and represented an overall conceptualisation of data patterns. The next stage of the process involved sorting the existing codes into potential themes. The use of mind-maps aided in the production of a thematic map that comprised candidate themes. Following this stage, themes were reviewed at the level of coded extracts to ensure the candidate themes adequately represented the coded data. Next, the validity of individual themes was considered in relation to the data set and whether candidate themes accurately represented the meanings evident in the whole data set. At this stage, themes were further defined and redefined. The first and second authors analysed the transcripts, coded extracts, and potential themes, any disagreements in interpretation were resolved through consensus. Themes were constructed at a semantic level, acknowledging concepts directly conveyed by participants, though consideration was afforded to possible latent concepts.

## Results

### Quantitative Results

Descriptive statistics provided the analyst with percentages of males and females within the study, mean age of participants, ethnicity, school role, duration within the profession, and any previous trauma training (see Table 1). The age of participants within the intervention sample ranged from 29 to 64 (*M* = 46.55, *SD* = 7.70). Females accounted for 71.4% of the sample. The sample consisted of 90.8% Caucasian. Most participants indicated their role within the school as teaching and learning support (77.6%) with participants indicating having been in the profession for over 15 years (71.4%) and having previously attended trauma related training (25%). The mean age of participants within the waitlist control group ranged from 22 to 66 (*M* = 41.91, *SD* = 11.02). Females accounted for 72.9% of the sample. The sample consisted of 86.2% Caucasian. Most participants indicated their role within the school as teaching and learning support (78.8%) with half of participants indicating having been in the profession for over 15 years (51.7%) and having previously attended trauma related training (13.6%).

## Attitudes Related to Trauma-Informed Care: Time 1 vs Time 2 Intervention Group

### Overall ARTIC

A paired-samples *t*-test (see Fig. 2) was conducted utilising intervention group data to compare overall levels of attitudes related to trauma-informed care reported by school personnel immediately before and again immediately following the two-day workshop (*n* = 75). There was a significant positive increase from pre- (*M* = 4.94, *SD* = 0.53), to post (*M* = 5.56, *SD* = 0.58) scores for overall ARTIC scores, *t* (74) = -11.70, *p* < 0.001, *d* = 1.35.

In addition, there was a significant positive increase in all ARTIC-35 subscales from pre to post training (see Fig. 2).

## Compassion Satisfaction/Fatigue Time 1 and Time 3 Intervention Group

**Fig. 3:**
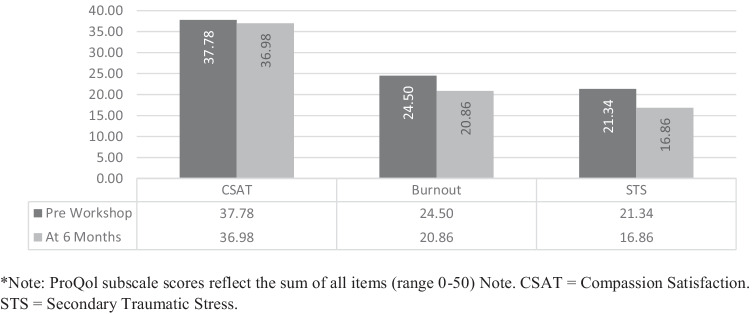
Time1/Time3 means of ProQol subscales of the intervention group (*n* = 50)

A paired-samples *t*-test (see Fig. 3) was conducted utilising intervention group data to compare levels of burnout reported by school personnel immediately before the two-day workshop and again at 6-month follow up (*n* = 50). There was a significant decrease from pre (*M* = 24.50, *SD* = 4.89), to post (*M* = 20.86, *SD* = 4.34) scores for burnout, *t* (49) = 7.51, *p* < 0.001, *d* = 1.06. Additionally, there was a significant decrease from pre (*M* = 21.34, *SD* = 5.63), to post (*M* = 16.86, *SD* = 3.77) scores for STS, *t* (49) = 8.60, *p* < 0.001, *d* = 1.21.

## Attitudes Related to Trauma-Informed Care: Time 1 and Time 3 Intervention Group

### Overall ARTIC

**Fig. 4:**
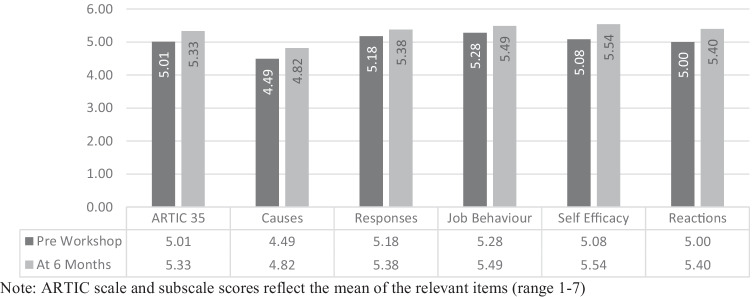
Time1/Time3 means of ARTIC scales pre- and 6-months following the workshop for the intervention group (*n* = 65)

A paired-samples *t*-test (see Fig. 4) was conducted utilising intervention group data to compare overall levels of attitudes related to trauma-informed care reported by school personnel immediately before training and again at 6-month follow up (*n* = 65). There was a significant positive increase from time one (*M* = 5.01, *SD* = 0.58), to time three (*M* = 5.33, *SD* = 0.63) scores for overall ARTIC scores, *t* (64) = -6.43, *p* < 0.001, *d* = 0.79.

There was also a significant positive increase in all ARTIC-35 subscales from pre-training to 6-month follow up (see Fig. 4).

## Intervention Versus Control Group (Time 1 v Time 3)

**Fig. 5:**
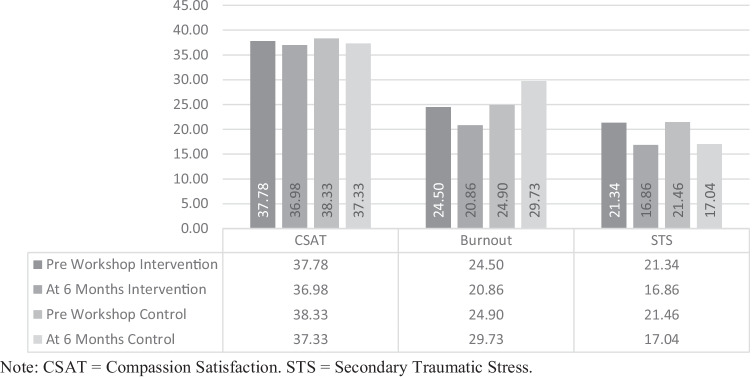
Pre-workshop and 6-month follow-up mean ProQol scale scores of the intervention and control groups

A 2 (time) × 2 (treatment) mixed ANOVA comprising of both intervention and waitlist control data revealed a significant time x treatment interaction effect for burnout *F* (1,100) = 69.09, *p* < 0.001, *η*_*p*_^*2*^ = 0.41 (see Fig. 5). Examination of cell means, and Bonferroni corrections indicated a significant reduction in burnout scores from time 1 (*M* = 24.50) to time 3 (*M* = 20.86) within the intervention group *t* (100) = 5.01, *p* < 0.001. In contrast, a significant increase in burnout scores from time 1 (*M* = 24.90) to time 3 (*M* = 29.73) within the control group was observed *t* (100) = 6.77, *p* < 0.001.

### Overall ARTIC

**Fig. 6:**
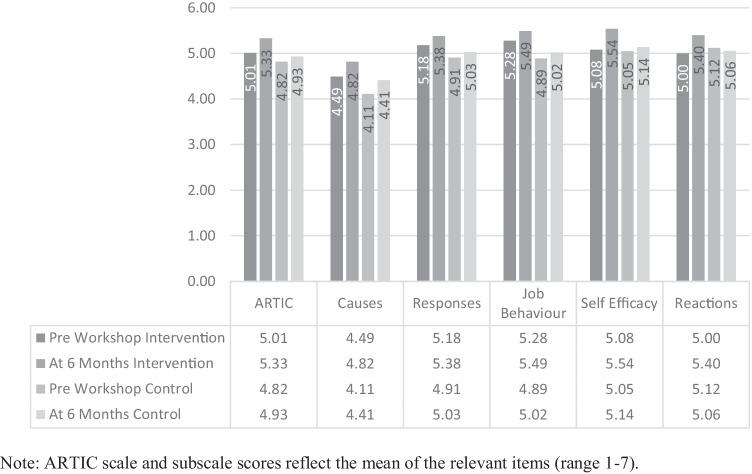
Pre-workshop and 6-month follow-up mean ARTIC scale scores of the intervention and control groups

A 2 (time) × 2 (treatment) mixed model ANOVA comprising of both intervention and waitlist control data revealed a significant time x treatment interaction effect for the overall ARTIC-35 scale *F* (1,113) = 4.56, *p* = 0.04, *η*_*p*_^*2*^ = 0.039 (see Fig. 6). Examination of cell means, and Bonferroni corrections indicated a significant increase in scores from time 1 (*M* = 5.01) to time 3 (*M* = 5.33) within the intervention group *t* (113) = 5.10, *p* < 0.001, and no significant change from time 1 (*M* = 4.82) to time 3 (*M* = 4.94) within the control group *t* (113) = 1.64, *p* = 0.10. Thus, when comparing time 1 to time 3, the intervention group demonstrated significantly higher ARTIC scores at six-month follow up whereas there was no change within the control group at this time.

### Self-Efficacy at Work

Next, the ANOVA revealed a significant time x treatment interaction effect for self-efficacy at work sub-scale *F* (1,113) = 6.66, *p* = 0.011, *η*_*p*_^*2*^ = 0.056. Examination of cell means, and Bonferroni corrections indicated a significant increase in scores from time 1 (*M* = 5.08) to time 3 (*M* = 5.54) within the intervention group *t* (113) = 4.78, *p* < 0.001. However, there was no significant change from time 1 (*M* = 5.05) to time 3 (*M* = 5.14) within the control group *t* (113) = 0.78, *p* = 0.44.

### Reactions to the Work

Further, ANOVA revealed a significant time x treatment interaction effect for reactions to the work subscale *F* (1,113) = 13.00, *p* < 0.001, *η*_*p*_^*2*^ = 0.10. Examination of cell means, and Bonferroni corrections indicated a significant increase in scores from time 1 (*M* = 5.00) to time 3 (*M* = 5.40) within the intervention group *t* (113) = 4.74, *p* < 0.001. However, there was no significant change from time 1 (*M* = 5.12) to time 3 (*M* = 5.06) within the control group *t* (113) = 0.07, *p* = 0.51.

### Qualitative Focus Group Findings

The teaching staff participating in the focus groups were interviewed by Dr Karen Kirby and Justin MacLochlainn of Ulster University. The purpose of the focus groups was to talk openly about teachers’ own experience following implementation of the Compassionate Schools framework. The data was derived from a semi-structured interview about their understanding following the training, how they felt about work-related changes following training, personal growth, barriers related to implementing the framework, self-care, and suggestions on how to improve the training. Participants were instructed to talk openly and at length on each topic. In order to protect anonymity teachers were coached to use pseudonyms. A bottom-up approach was used, and the themes emerged from the coding of the data. The four themes that arose from the data were, *Challenges*, *Self-Care*, *Outreach*, and *Role adaptation*. This section will demonstrate with the use of selected extracts how these themes relate back to the teachers’ experience following implementation of the Compassionate Schools framework.

### Challenges

Participants were asked if any barriers were present to implementing any aspect of the CS framework in the school. Joan was first to respond and immediately conveyed the pressures teaching staff were operating under such as, getting grades, percentages up, all the while working hard with trauma-impacted youth to improve outcomes for these students:

Joan: (clears throat) “*We still, we still have to get results, we still have to get GCSE grades, we still have to get up percentages, we still have to work hard with kids who maybe, the trauma is impacting on coursework and exams results and that kind of thing”.*

Applying a deeper systemic analysis, Dave points out that the conditions teaching staff were currently operating under were caused by political decisions such as budget cuts and class size increases:

Dave: “*I do think just the pressures that are on staff, all staff, is continually, just year by year just increasing, and it’s nothing to do with anything else apart from (pause) budget cuts, higher class numbers, all the usual things and it’s, it’s stuff that’s been forced upon you, you know* ….”.

Emily confirmed that teachers were struggling with finding solutions to classroom disruption and struggling with implementing the behaviour management policy and called for more support from senior leadership within the school:

Emily: *“…. erm, as a whole school approach, I find that we’re still struggling, we’re still struggling to find what works and what doesn’t work, you know certainly erm, staff are trying their very, very best but I think the support of the behaviour management and things like that maybe isn’t as firm and strong as it could be*”.

The above extracts underline the theme “Challenges” illustrated by the pressure teaching staff within post-primary schools are operating under in N.I. Some staff felt disempowered in the face of relentless austerity imposed by educational policy, whereas others alluded to the perceived lack of support from senior leadership in implementing the behaviour management policy.

### Self-Care

Next, participants were asked what they had, or what the school had introduced as strategies to prevent or alleviate staff stress. Zoe indicated support from her peers but alludes to feelings of abandonment when she implies that senior management were not taking self-care of their staff seriously:

Zoe: “*I think we help each other but further up I don’t think they see us; they see us with getting on with our work or getting on with, you know, the reports you’re marking or your grades… (interrupted)”.*

Chris interrupts with some perceived discontent aimed towards senior management regarding care of teaching staff:

Chris: “…...*there was one occasion where we were told to go for self-care for half a day, but we were told on that day, so you can’t really organize anything or, you know, it was great to get it but it would be nice to be told in time so you could plan something*”.

Bernadette explains how her department safeguards teaching staffs’ health by meeting up and talking through any ongoing concerns:

Bernadette: “*Erm, well we are in a different department so we are you know, we would help each other, we always talk at break, at lunch and that kinda thing, you know talking is great erm, yeah we have a supportive department which I think really helps*”.

Overall, the theme “Self-Care” identified pressures relating to the administrative duties, classroom duties, and perceived lack of communication from senior leadership was impacting teaching staffs’ ability to practice self-care. However, some departments within the school may have been better equipped to provide staff with the space to cooperate and support each other than others.

### Outreach

Following this, participants were asked whether there was anything else that they (participants) would like to add that would assist teaching staff establish a trauma-informed classroom? John opened expressing some frustration because of students who he perceived as not being invested in trauma informed practice. Teaching staff have bought into whole school trauma informed practice; however, students have not received any CS training:

John: “*I’d be interested in the pupils voice because we are, we are buying into it and I think it’s frustrating because you’re trying to be more patient more restorative, more trauma-informed and you’re doing your bit and you do feel let down if they (students) don’t buy into it”…..”you kinda, you kinda expect maybe that the penny will drop with them at some point”.*

The above observation was supported by Emily who suggested that everybody within the school needed to understand what it meant to be a trauma informed school. At present, students were confused and did not understand what trauma informed CS was about.

Emily: *“It needs to be everybody that has a better understanding, you know, the kids don’t understand what a trauma-informed compassionate school is, and they’re kinda going well what’s this all about….”.*

To clarify what was said previous, Interviewer 1 asked. “So, you think students need to be more aware themselves of everything about trauma informed schools”?

Cora: “*Yes” (all nodded in agreement and said “yes”).*

Admitting that training students in the CS approach was beyond the skills of teaching staff within the school, Cora asked for workshops to be delivered for students from outside agencies:

Cora: “*Because we don’t have the skills for that, we need help from people to come in and take our students through …... workshops”.*

Jodie interrupts by asserting the importance of workshops for students and included the need for booster workshops for staff in recognition that being trauma-informed requires more than just once off training. She suggests ongoing workshop training for students too:

Jodie: *“Aye, yes, that is very important, the workshops and everything else. erm, we would need backup after those as well, it’s not just a one-off workshop, it’s definitely, that there’s somebody that comes in weekly or whatever length of time (interrupted)”.*

Picking up on this comment, Emily indicated some parental and community confusion surrounding what it means to be trauma informed. She reasoned parents viewed CS as ‘just another thing’ that the school had introduced, and in doing so, inferred a lack of dissemination of the framework to parents from the school. She goes further to endorse trauma-informed training for parents similar to what staff at the school received. This, she believes, would provide a better, rounded, understanding of what it means to be a trauma-informed school:

Emily: “…..*parents often don’t, they just see it as another thing that the school does, erm, so I think maybe a bit more training you know training for the kids on a level for them erm, and then bring parents, and bring parents in and do the training that the staff have received as well, you know, just so that everybody is aware of, well look this is what it means this is what we’re trying to do erm, and then there’s a better understanding all around…”.*

In summary, the theme ‘Outreach’ incorporated two well-defined groups: students and parents. Some teaching staff recognised that students were not invested in the CS program so put forward recommendations for outside facilitators to come into school and deliver ongoing workshops to students to assist their understanding.

### Role Adaptation

Next, participants were asked about their understanding of how trauma impacts learning and whether their (participants) attitudes had changed in any way. Dave opened and asserted that the CS training made him more aware of learning difficulties students were facing in his classroom. Furthermore, Dave was ‘seeing’ more trauma-impacted students in his classroom because of his increased awareness following training. His empathy for trauma-impacted students transformed to a compassionate approach as he attempts to help these students in need.

Dave: “ W*e really see how it effects barriers to learning, how it affects them”…..”and learning difficulties as well, I mean all that there really hit home”…..”you are more aware now, you are looking out for more students, and you are, you are seeing more students who’s affected and then you’re sorta zoning in on them and trying to help you know*”.

Focusing on specific components of the CS training, Zoe derived most benefit from the training encompassing the fight/flight/freeze/fawn responses. The CS training provided her with the knowledge to recognise dysregulated behaviours in the classroom along with advancing more compassionate approaches to these behaviours:

Zoe: *“…. the fight and flight kind of idea, em, I think that was really beneficial within the classroom, just when the child was coming in with trauma, you know, just to even know the way in which they’re acting is, this is the part of them going through this process you know…..”*

Emily goes further to summarise what she felt the CS training afforded teaching staff. She acknowledged the need to adapt previous approaches to misbehaviour to a more inclusive, supportive, and compassionate role in the classroom:

Emily: “I*t’s just all about changing our approach to how we deal with the students, you know, it’s looking beyond the behaviour and asking why, why is the behaviour like this? And what can we do to support the pupils erm, in, in behaviour and the type of behaviour and you are just more compassionate, and you try to be more understanding towards, you know (interrupted)”.*

To elucidate whether any further behaviour change in response to the training had taken place among participants, Interviewer 2 asked, “What have you changed in response to the training”? Zoe explained that she now takes a little time to reflect on what would be the best response to the current situation, and in doing so, avoids knee-jerk reactions:

Zoe: “I*t’s about taking that pause and realising I’m not reacting and kinda going right, what’s going on here and weighing up the whole situation and then responding in the appropriate way just”.*

Adapting to a more trauma-focused compassionate perspective, Kevin used language to connect and build relationships with his students:

Kevin: “….*again it’s about the language we use, not what’s wrong but what’s happened, what’s happened for you to behave like this, so it’s changed my way of thinking in that sense”.*

Finally, Joan recognised an increase in her awareness of the difficulties trauma-impacted students may be experiencing in the classroom in response to CS training. Furthermore, she believed that the training had assisted her in being able to ‘cope’ with situations as they arose:

Joan: “*I think it’s made me more aware of what the children are going through, you know, just how to approach them and you know, just being very, very aware of that, you know, how they may react, you know, and what way for as a teacher to cope with that particular situation…...” (all nodded in agreement).*

In sum, the above extracts demonstrate that the trauma-informed CS training workshop had a positive effect on teaching staff perspectives, attitudes, and behaviours, which led teachers to adapt their roles within the classroom to a more trauma-informed, compassionate approach.

## Discussion

The increasing awareness of the ubiquitous nature and detrimental effects of adverse childhood experience, stress, and trauma has ignited interest in trauma-informed care across service sectors (Kenny et al., 2017). Within schools, teachers have reported that stress resulting from students’ disruptive behaviour as being central to experiences of burnout (Fazel et al., 2014). However, teachers have limited training, knowledge, or skills, to recognise student misbehaviour as reactions to toxic stress and trauma, and therefore are often locked in negative cycles of punitive approaches and escalating misbehaviour. Providing whole-school trauma-informed care (TIC) training is perceived as a practical way to disrupt these negative cycles by attempting to inform staff on the nature of trauma and in doing so, change attitudes toward trauma-impacted students.

The primary aims of the current study were to determine whether a 2-day professional development training programme in trauma-informed compassionate schools (Wolpow et al., 2009) would lead to changes in school personnel attitudes towards trauma-informed care and that these changes would be maintained at 6-month follow up. Additionally, the study set out to determine whether the implementation of trauma-informed CS within the school influenced school personnel levels of compassion satisfaction, secondary traumatic stress, and burnout. To ensure any significant findings were robust, the study employed a waitlist control group yielding a between-groups comparison. The control schools had similar student numbers and are both post-primary schools situated in N.I.

With the use of a mixed methods approach, a fuller understanding of the complexities involved in the implementation of the programme surfaced. Within this study, both quantitative and qualitative data supported the positive impact of CS training on teacher attitudes towards trauma-impacted students. Regarding quantitative findings, the study evaluated measures on attitudes related to trauma-informed care pre-, and immediately following the professional development training within the intervention group. Despite the fact this group showed baseline scores on the overall ARTIC scale and subscales that were above the midpoint (i.e., participants were already endorsing trauma-informed attitudes before training leaving comparatively little room for improvement), findings revealed the 2-day compassionate schools training programme had an immediate, positive, significant effect on attitudes related to trauma-informed care.

Next, the study evaluated whether any change from pre-workshop ARTIC scores within the intervention group to the 6-month follow up scores took place. Findings showed a positive change in the overall ARTIC and all subscales, suggesting that participation in the workshop had experienced positive and lasting effects on attitudes related to trauma-informed care. These findings corresponded with the theme of *Role Adaptation* developed from focus group interviews. Participants agreed that they were now more aware of the profound impact trauma can have on their students, they also believed training had assisted them in their ability to cope with dysregulation in the classroom.

Following this, the study determined whether the implementation of trauma-informed CS within the school influenced school personnel levels of compassion satisfaction (CSAT), secondary traumatic stress (STS), and burnout within the intervention group. Results showed no change in CSAT but a significant decrease in STS and burnout as measured at 6-month follow up. Baseline scores indicated that the intervention group had already moderate levels of CSAT, STS, and burnout. At 6-months, levels of STS and burnout decreased to low, thus conceivably demonstrating the efficacy of the combined workshop modules, and in particular, the modules pertaining to self- care.

It should be noted that following eliciting the themes of *Challenges*, and *Self-care* from the focus group interviews, the researchers immediately brought forward the concerns of participants to the school leadership team via a whole-school booster session on classroom management and self-care strategies. Teaching staff concerns around systemic issues, and perceived lack of senior leadership support were voiced during this session with senior leadership and teaching staff resolving the behaviour management policy to be more inclusive and supportive of a whole school approach, thus amendments were made to ensure classroom support was being provided to all teaching staff and that this was led by senior management (Muijs & Harris, 2006). Strategies of self-care for teaching staff were also agreed upon as per recommendations set out within the CS framework. Self-care strategies are comprised of physical, emotional, cognitive, social, and spiritual self-care. School personnel were made aware that self-care was an ethical responsibility and were instructed how they can avert or diminish the impact of STS and burnout in the classroom. Understanding how to prevent burnout by prioritizing self-care in addition to senior leadership buy-in may have ameliorated feelings of exhaustion, frustration, anger, and depression in participants within the intervention sample.

Finally, the study compared the intervention group with the waitlist control group on all measures pre-workshop and at 6-month follow up. When comparing time 1 to time 3 of both groups, the intervention group demonstrated higher overall ARTIC, self-efficacy at work, and reactions to the work subscale scores at six-month follow up, whereas there was no change within the control group at this time. In comparison to the control group, burnout levels of the intervention group went from moderate to low at 6-months, whereas control levels of burnout remained within the parameters of moderate though did increase.

A possible causal interpretation of the quantitative findings above may be that since self-efficacy (i.e., self-evaluation and self-perception) is derived from positive peer support and strong school leadership, staff may have averted increases in STS and burnout due to buy-in from school leadership (Skaalvik & Skaalvik, 2010). Supportive school leadership provides staff with shared norms, goals, and values which may increase staff beliefs of the ability of the school to execute courses of action necessary to produce desired outcomes (Muijs & Harris, 2006). With school leadership buying into the CS paradigm espousing high expectations of classroom management and staff self-care, staff self-efficacy has begun to flourish and in turn lessening symptoms of burnout.

In relation to the focus group theme *Outreach*, some teaching staff recognised that students were not invested in the CS program and put forward recommendations for outside facilitators to come into school and deliver ongoing workshops to students to assist their understanding. This strategy to educate students on the deleterious effects stress and trauma have on the developing brain is not unique, however, very rare within the research literature. Carello and Butler (2014) suggest that the risk of re-traumatisation and secondary traumatisation should be decreased rather than increased and go on to propose that any trauma-informed approach to pedagogy should recognise these risks and promote students’ emotional safety first and foremost (Carello & Butler, 2014). Overall, focus group responses demonstrated that participants were broadly supportive and accommodating of the CS framework as evidenced by recommendations from the group that CS training should also be undertaken with both student and parent cohorts.

These findings replicate previous studies evaluating the CS programme in an educational setting (Parker et al., 2019), and add to the small yet growing evidence-based research on TIC implementation using theoretical grounded, effective TIC models in schools. Furthermore, and to our knowledge, this study is the first study evaluating TIC programmes in schools that establishes internal validity by way of a control group. Bolstered by this quasi-experimental design, these findings demonstrate that when school personnel are provided with psychoeducation that they were unlikely to receive in pre-service training, it had an immediate and long-term impact on attitudinal change towards their trauma-impacted students. Modules of the CS training comprising of neuroscience, neurobiology, and psychology, enabled school personnel to recognise and understand ACEs, stress, and trauma and how these affect students’ biological, psychological, and social well-being. Additionally, instruction on self-care and on recognising and responding to stress and how stress manifests in the classroom along with school leadership buy-in seemed to de-escalate patterns of burnout in participants within the intervention group. Finally, teaching staff advocating for and endorsing CS training represents preliminary but encouraging evidence of the suitability and acceptability of CS training in schools (Parker et al., 2019).

## Limitations

Our overall findings should be considered in light of several limitations. The quantitative study relied entirely on self-report questionnaires and are consequently subject to an array of concerns regarding that form of data-collection. Furthermore, the ARTIC scale is a measure of attitudes and not behaviours, therefore, positive results cannot be translated to real-world actions of school personnel. Future studies should attempt to connect attitudinal change with real-world behavioural change of personnel and endeavour to correlate these changes to improvements in student behaviour and academic attainment. Moreover, this study was unable to dismantle, or portion components of the 2-day training workshop. For instance, was every module presented at training necessary to create the change reported, or were some modules demonstratable more valuable than others? Could some modules be excluded with no apparent effect on outcomes? Which modules were most predictive of attitudinal change, and change in levels of STS and burnout? Future studies should investigate these questions to improve the delivery and enhance the paradigm as well as evaluate its impact. Indeed, conferring directly with young people within the school system about the changes being made may be an important indicator to the success of any trauma-informed intervention, therefore an important factor to include in future research. Reporting on the impressions of students along with organisational and systemic outcomes is a useful form of triangulation and an avenue for future investigation.

## Recommendations

While most educators receive little training in recognising the signs and symptoms of primary traumatic experience in their student population, they receive no training in the self-care necessary to prevent compassion fatigue in themselves (Wolpow et al., 2009). In response to insufficient training of school staff, school leadership should be engaged in activities to promote organisational culture, policies, and practices to support staff. These recommendations comprise of placing focus on prevention by being proactive in addressing stress management, reinforcing natural support systems for school personnel, and continuous evaluation of ongoing efforts to ameliorate compassion fatigue (SAMHSA, 2014). School administrators should shoulder responsibility for embedding practices that promote self-care for all school personnel who are frontline staff dealing with trauma and adversity (Thomas et al., 2019). The implementation of trauma-informed care is a top-down system-level intervention that seeks to transform the environment of service provision, to embed a more empathetic and compassionate culture, and define policy to ensure the risk of re-traumatisation is minimised (Lowenthal, 2020). In addition, TIC in the education system should not be viewed as a short-term fix as success relies on adequate groundwork being laid to guarantee genuine buy-in. Success will also depend on sufficient resources, both financial and human, to be released to ensure a shift in paradigm. Including community partnerships ensure that these approaches gain a foothold and the benefits accrued can be maximised (Bunting et al., 2019).

Moreover, the approach of delivering interdisciplinary knowledge employing outside specialists to teach school staff on a one off or intermittent basis is one that is surely unsustainable. With rates of teacher turnover increasing, schools are at risk of hiring new teachers that have little to no training in trauma-informed practice. Therefore, it is recommended that school administrators at state level advocate for teacher pre-service programs to comprise of well-grounded, and methodologically rigorous research and practice that compels changes in teaching practice towards a trauma-informed approach. This approach will both, guide teachers to recognise their unique role and accept their responsibilities to improve the outcomes of trauma-impacted youth and support the growth of ‘whole-school’ implementation going forward.

Ultimately, for any school to authentically embed trauma informed principles, that schools’ discipline or behaviour management policy may need to be revised. This was observed within the intervention school where it was clear that the trauma-informed principles would not be acted upon or taken forward unless the discipline policy was revised to reflect trauma informed principles and strategies; for example, removing suspension and expulsion as punishments, and considering choice and consequences instead (five acts of kindness rather than detention). We found that proposed revisions had to be carried out in collaborative way, involving both students, teachers and senior management who met, discussed and revised the discipline policy.

In addition to this, the intervention school joined more effectively with other outside supportive agencies; for example, when children disclosed to teachers that they were experiencing trauma at home or in the community, the intervention school increased their engagement with the social work gateway and family intervention services, in addition to other key workers from agencies such as ‘Start 360’ who support families/parents who struggle with addiction. Finally, the intervention school developed a better relationship with the local CAMHS Psychologist who provided additional supportive consultations as an when required throughout the intervention.

## Conclusion

This study was the first whole-school trauma-informed professional development training workshop that utilised more robust research methods by the means of a control group. This study contributes to the empirical evidence relating to trauma-informed approaches in schools. The study was designed to determine attitudinal change of school staff following implementation of a whole-school trauma-informed intervention (workshop). The content of the workshop material provided the framework for the development of a more compassionate, supportive, safe classroom environment in addition to improving teacher well-being (Wolpow et al., 2009). Our findings demonstrate that with minimum training on the dynamics of trauma, all personnel attached to a school can become more trauma-informed and as such, have more favourable attitudes towards trauma-impacted students and consequently be less likely to experience burnout. These findings support the ongoing evaluation of the CS paradigm as a potential framework for ameliorating the negative impact of trauma and burnout and contributes to the small but growing body of research in promoting more trauma-informed attitudes and improving staff wellness in schools.

## Data Availability

On request.

## Code Availability

N/A.
